# Arginine alleviates *Clostridium perfringens* α toxin-induced intestinal injury *in vivo* and *in vitro* via the SLC38A9/mTORC1 pathway

**DOI:** 10.3389/fimmu.2024.1357072

**Published:** 2024-04-04

**Authors:** Xiaohui Wang, Tong Zhang, Wenli Li, Heliang Wang, Lei Yan, Xiaowen Zhang, Lianwen Zhao, Nianxue Wang, Beibei Zhang

**Affiliations:** ^1^College of Animal Science and Technology, Qingdao Agricultural University, Qingdao, China; ^2^Qingdao Sino-science Gene Technology Co., Ltd, Qingdao, China; ^3^Shandong New Hope Liuhe Group, Qingdao, China

**Keywords:** arginine, *Clostridium perfringens* α toxin, intestinal inflammation, cell apoptosis, SLC38A9/mTORC1 pathway, broiler chickens, IEC-6 cells

## Abstract

**Introduction:**

*Clostridium perfringens* α toxin is a main virulence factor responsible for gut damage in animals. Arginine is a functional amino acid exhibiting significant immunoregulatory activities. However, the effects and immunoregulatory mechanisms of arginine supplementation on α toxin-induced intestinal injury remain unclear.

**Methods:**

*In vivo*, 256 male Arbor Acres chickens were randomly assigned to a 2×2 factorial arrangement, involving diet treatments (with or without 0.3% arginine supplementation) and immunological stress (with or without α toxin challenge). *In vitro*, IEC-6 cells were treated with or without arginine in the presence or absence of α toxin. Moreover, IEC-6 cells were transfected with siRNA targeting mTOR and SLC38A9 to explore the underlying mechanisms.

**Results and discussion:**

The results showed that *in vivo*, arginine supplementation significantly alleviated the α toxin-induced growth performance impairment, decreases in serum immunoglobulin (Ig)A and IgG levels, and intestinal morphology damage. Arginine supplementation also significantly reduced the α toxin-induced increase in jejunal proinflammatory cytokines interleukin *(IL)-1β*, *IL-6* and *IL-17* mRNA expression. *Clostridium perfringens* α toxin significantly decreased jejunal mechanistic target of rapamycin *(mTOR)* and solute carrier family 38 member 9 (*SLC38A9)* mRNA expression, while arginine supplementation significantly increased *mTOR* and *SLC38A9* mRNA expression. *In vitro*, arginine pretreatment mitigated the α toxin-induced decrease in cell viability and the increase in cytotoxicity and apoptosis. Arginine pretreatment also alleviated the α toxin-induced upregulation of mRNA expression of inflammation-related cytokines *IL-6*, C-X-C motif chemokine ligand (*CXCL*)*10*, *CXCL11* and transforming growth factor-β (*TGF-β*), as well as apoptosis-related genes B-cell lymphoma-2 associated X protein (*Bax*), B-cell lymphoma-2 (*Bcl-2*), B-cell lymphoma-extra large (*Bcl-XL*) and cysteinyl aspartate specific proteinase 3 (*Caspase-3*) and the ratio of *Bax* to *Bcl-2*. Arginine pretreatment significantly increased the α toxin-induced decrease in *mTOR*, *SLC38A9*, eukaryotic translation initiation factor 4E (eIF4E)-binding protein 1 (*4EBP1*) and ribosomal protein S6 kinase (*S6K*) mRNA expression. Knockdown SLC38A9 and mTOR largely abrogated the positive effects of arginine pretreatment on α toxin-induced intracellular changes. Furthermore, SLC38A9 silencing abolished the increased *mTOR* mRNA expression caused by arginine pretreatment. In conclusion, arginine administration attenuated α toxin-induced intestinal injury *in vivo* and *in vitro*, which could be associated with the downregulation of inflammation via regulating SLC38A9/mTORC1 pathway.

## Introduction

*Clostridium perfringens*, a Gram-positive, anaerobic, opportunistic pathogen, is ubiquitous in diverse environments, including in soil, sewage, and the gastrointestinal tracts of humans and animals (1). *C. perfringens* has been implicated in a wide range of serious diseases in muscle, the gastrointestinal tract, and other organs and tissues. These disorders include gas gangrene, necrotizing enteritis, enterotoxemia, foodborne or non-foodborne poisoning of diarrhea, and necrotic enteritis (2). Necrotic enteritis is a gastrointestinal disease in poultry, exhibiting in two forms: acute and chronic. The acute form is characterized by anorexia, severe morbidity, and substantial mortality. The chronic form is marked by slowed growth performance, lowered feed efficiency, and lethargy (3). *C. perfringens* α toxin is recognized as a key virulence factor in the pathogenesis of avian necrotic enteritis (4–6). This toxin exhibits phospholipase C and sphingomyelinase activities, resulting in extreme cytotoxicity and myotoxicity. Furthermore, it can induce hemolysis, necrosis, damage to cell membranes, the generation of superoxide radicals and inflammatory cytokines (7–10).

L-arginine is a functional amino acid in both humans and animals, serving as an important precursor for the synthesis of nitric oxide, polyamines and creatine during immune responses. Numerous studies have highlighted the significant benefits of L-arginine in preserving intestinal health across various disease models (11–13). Zheng et al. (13) reported that arginine supplementation exerted beneficial effects on the intestine by regulating arginine metabolism and reducing inflammatory cytokine expression in piglets under oxidative stress. Lan et al. (11) discovered that L-arginine effectively alleviated intestinal inflammation induced by lipopolysaccharide by inhibiting the TLR4/NF-κB and MAPK pathways while enhancing β-Defensin expression both *in vivo* and *in vitro*. Moreover, our previous study demonstrated that dietary arginine supplementation alleviated serum arginine depletion induced by *C. perfringens* challenge, restored normal arginine transport and catabolism in the intestine, mitigated the inflammatory response, and suppressed the JAK-STAT signaling pathway in broiler chickens, thereby safeguarding their intestinal health (14).

Mechanistic target of rapamycin complex 1 (mTORC1) is a conserved multi-protein complexes sensitive to changes in intracellular levels of amino acids, glucose and lipids, as well as extracellular stimuli such as cytokines, growth factors, and Toll-like receptors (15). It plays a critical role in regulating essential physiological functions, including protein synthesis, cellular metabolism, immune responses, inflammatory reactions, and apoptosis (16, 17). Arginine is known to promote diverse physiological effects largely through the activation of mTORC1 (18–20). Recent discoveries have identified solute carrier family 38 member 9 (SLC38A9) as an arginine sensor located upstream of mTORC1, thereby positively regulating the mTORC1 signaling pathway (21–23). Through the activation of mTOR, arginine triggers its downstream targets, including eukaryotic translation initiation factor 4E (eIF4E)-binding protein 1 (4EBP1) and ribosomal protein S6 kinase (S6K), which play key roles in protein synthesis (24, 25) and the modulation of intestinal inflammation (26). However, whether the SLC38A9/mTORC1 signaling pathway plays an important role in the protective effects of arginine against α toxin challenge remains unclear.

Therefore, this study was conducted to determine the effects of arginine administration on intestinal injury induced by the α toxin challenge *in vivo* and *in vitro*. Further, the possible immunoregulatory mechanism based on the SLC38A9/mTORC1 pathway was explored. This research contributes to the understanding of nutritional interventions for enhancing host resilience to pathogenic challenges.

## Materials and methods

### Experimental design and animals

The experiments were approved by the Animal Care and Use Committee of Qingdao Agricultural University (No. DKY20220905). The experimental design followed a 2×2 factorial arrangement. A total of 256 one-day-old male Arbor Acre broilers with similar initial body weight were selected and categorized into four groups. The groups were as follows: CON group, birds fed a basal diet without α toxin challenge; ARG group, birds fed a basal diet supplemented with 0.3% arginine without α toxin challenge; ATX group, birds fed a basal diet and subjected to α toxin challenge; ARG+ATX: birds fed a basal diet supplemented with 0.3% arginine and subjected to α toxin challenge. Each group was divided into 8 replicates, each containing 8 chickens. At 15, 17, 19 and 21 days of age, the birds were intraperitoneally injected with α toxin (Sigma, P7633; in groups ATX and ARG+ATX) at a dosage of 0.1 U/kg of body weight or an equal volume of phosphate-buffered saline (PBS; in groups CON and ARG). The experimental diet was made in mash form and formulated in accordance with the China feeding standard of chicken (NY/T 33-2004). The diet composition and nutritional levels are provided in **Supplementary Table 1**. L-arginine with a purity of 98.5% was purchased from CJ CheilJedang Corporation in South Korea, while L-alanine with a purity of 98% was obtained from Anhui Huaheng Biotechnology Co., Ltd. The room temperature was maintained at 33°C for the first week and gradually decreased to 26°C in the third week. The relative humidity was controlled at 50%~70%. The experiment was conducted using a three-tier cage system with 23 h of light and 1 h of darkness each day. The broiler chickens had ad libitum access to feed and water. Standard management and immunization procedures were employed throughout the experimental period.

### Growth performance

On d 14 and d 21, feed consumption and body weight for each replicate were assessed. Average daily gain (ADG), average daily feed intake (ADFI), and feed conversion ratio (FCR) were calculated for the periods from d 1 to 14 and from d 15 to 21.

### Sample collection

On the 21 d of age in broiler chickens, 3 h after toxin injection, one chicken was randomly selected from each replicate. Aseptic blood samples were drawn from the bird’s wing veins, and serum was obtained by centrifugation for 10 min at 3000 r/min at 4°C. The serum was kept for further analysis at -20°C. Subsequently, the chicken was euthanized by exsanguination from the neck vein, and the intestines were isolated. A small section from the midsection of the jejunum was collected and fixed in a 4% paraformaldehyde solution for the preparation of hematein-eosin-stained slides. Furthermore, tissue from the midsection of the jejunum was collected, rapidly frozen in liquid nitrogen, and kept at -80°C for gene expression level determination.

### Measurement of serum immunoglobulins (Ig) levels

The levels of IgA (M1244L96), IgG (M1245L96), and IgM (M1246L96) in the serum were measured using chicken ELISA kits (Shanghai Meilian Biological Engineering Co. Ltd., Shanghai, China), following the manufacturer’s instructions.

### Intestinal morphology analysis

Jejunal segments were immersed in a 4% polyformaldehyde phosphate buffer for 48 h to ensure proper fixation. The segments were dehydrated, embedded in paraffin, and sectioned to 4 μm thickness before being stained with hematoxylin and eosin. The microscopic images were captured using a Leica model DMi8 microscope (Leica, Wetzlar, Germany) and analyzed with image analysis software (version 4.2, Leica Application Suite, Leica, Wetzlar, Germany). Villus height was measured from the apex of the villus to the junction of the villus and the crypt. Crypt depth was determined as the vertical distance from the base of the crypt up to the junction of the villus and crypt, and the villus height-to-crypt depth ratio (VCR) was then calculated. Ten complete villi and their associated crypts were determined in each section at a magnification of 50×.

### Real-time quantitative PCR

Total RNA was extracted using TRIzol reagent (Invitrogen Life Technologies, Carlsbad, California, USA). The concentration and purity of RNA samples were determined using an NanoPhotometer NP80 (Implen, München, Germany). Subsequently, 1 μg of total RNA was reverse transcribed into cDNA using the PrimeScript™ RT reagent kit with gDNA Eraser (Takara Bio Inc., Dalian, China). The systhesized cDNA was stored at -20°C for further use. Quantitative PCR was performed to analyze the mRNA expression of target genes. Primers were synthesized by Shanghai Sangon Biotech Co., Ltd. Quantitative PCR was performed using the TB Green^®^ Premix Ex TaqTM (Takara Bio Inc., Dalian, China) following the manufacturer’s instructions. The amplification was carried out on a CFX96 Real-Time PCR Detection Systems (Bio-rad, Hercules, CA). The PCR protocol included an initial denaturation at 95°C for 30 seconds, followed by 40 cycles of denaturation at 95°C for 5 seconds and annealing/extension at 60°C for 30 seconds. The relative gene expression levels were calculated using the 2^-ΔΔCT^ method with *GAPDH* as the reference gene. The primer sequences for the target and reference genes *in vivo* and *in vitro* are presented in **Supplementary Tables 2**, **3**, respectively.

### Cell culture and treatment

The rat small intestinal epithelial cell line IEC-6 was acquired from Peking Union Medical College (Beijing, China) and cultured in Dulbecco’s Modified Eagle Medium (DMEM, Hyclone, Logan, UT) containing 5% (vol/vol) fetal bovine serum (Gibco, Carlsbad, CA), 100 U/mL penicillin, 100 mg/mL streptomycin (Solarbio, Beijing, China) and 0.01 mg/mL bovine insulin (Solarbio, Beijing, China). The cells were incubated at 37°C with 5% CO_2_. Upon reaching 80% confluence, the cells were divided into 4 groups: Con group (cells cultured in arginine-free DMEM without α toxin), Arg group (cells cultured with 4 mM arginine without α toxin), Tox group (cells cultured in arginine-free DMEM followed by 50 U/L α toxin incubation, and Arg+Tox group (cells cultured with 4 mM arginine for 24 h, followed by incubation with both 50 U/L α toxin and 4 mM arginine for an additional 4 h).

In the RNA interference experiment, the primer sequences for small interfering RNA (siRNA) targeting mTOR were as follows: 5’-GGCAUAUGGUCGAGAUUUATT-3’ and 5’-UAAAUCUCGACCAUAUGCCTT-3’. The primer sequences for siRNA targeting SLC38A9 were as follows: 5’-GGCUCUGCCUAUAAACUUATT-3’ and 5’-UAAGUUUAUAGGCAGAGCCTT-3’. The siRNAs were transfected into IEC-6 cells using Lipofectamine 3000 reagent (Invitrogen, Carlsbad, CA, USA) according to the manufacturer’s instructions. Subsequently, the culture medium was replaced with arginine-free DMEM, and the cells were treated with either 0 or 4 mM arginine for 24 h, followed by α toxin or PBS treatment for another 4 h.

### Cell viability assay

Cell viability was evaluated using the Cell Counting Kit-8 (CCK-8) test kit (Dojindo, Japan). In 96-well culture plates, approximately 1 × 10^4^ cells were seeded per well and incubated in complete medium for 36 h. Subsequently, the cells were washed twice with PBS. IEC-6 cells were pre-treated with either 0 or 4 mM arginine for 24 h, followed by treatment with PBS or 50 U/L of α toxin. After 3 h of α toxin treatment, the CCK-8 reagent was added to each well and incubated for 1 h at 37°C. The absorbance was determined using a microplate reader (TECAN, Infinite M Nano, Männedorf, Switzerland) at 450 nm. The absorbance values were normalized to those of the Con group.

### Lactic dehydrogenase (LDH) activity assay

LDH activity in the cell culture supernatants was determined to evaluate cytotoxicity using a commercially available LDH assay kit (Beyotime Biotechnology, Beijing, China) following the manufacturer’s protocol.

### Flow cytometry analysis

The Annexin V-FITC/propidium iodide (PI) apoptosis detection kit (Dojindo, Japan) was used to determine the rate of cell apoptosis following the manufacturer’s instructions. Cells were cultured in 12-well plates at a density of 1×10^5^ cells per well for 72 h. Subsequently, the cells were treated with either 0 or 4 mM arginine, followed by PBS or α toxin exposure. After treatment, the cells were harvested, washed twice with PBS, and resuspended in Annexin V binding solution to make a cell suspension with a final concentration of 1×10^6^ cells/mL. FITC-labeled Annexin V and PI were added to the cell suspension, followed by an incubation in the dark at room temperature for 15 minutes. An additional Annexin V binding solution was then added, and the samples was analyzed within 1 h using a flow cytometer (BD Bioscience, BD FACS Calibur, USA).

### TUNEL assay

Approximately 1×10^4^ cells were seeded per well in 96-well culture plates and incubated for 36 h. After treatment with arginine and α toxin, cell apoptosis was evaluated using the One Step TUNEL Apoptosis Assay Kit (Beyotime, Shanghai, China) according to the manufacturer’s instructions. The cells were observed under a fluorescence microscope (DMi8; Leica Microsystems, Wetzlar, Germany), and four random fields in each well were selected for cell counting using Image Pro Plus software.

### Statistical analysis

In the *in vivo* experiment, the general linear model procedure of SPSS version 25.0 (SPSS Inc., Chicago, IL) was used to evaluate the main effects of arginine supplementation, α toxin challenge, and their interaction. If a significant interaction effect was observed, one-way ANOVA and Duncan’s multiple comparison were used to examine the differences across the groups. For the *in vitro* experiment, data between two groups were analyzed by Student’s *t* test. Statistical significance was considered at *P*< 0.05. Graphs were generated using GraphPad Prism 8 Software (GraphPad Software Inc., La Jolla, CA, USA), with error bars representing the standard error of the mean (SEM).

## Results

### Arginine alleviated α toxin-induced growth performance impairment in broiler chickens

As shown in **Table 1**, no changes were observed in the ADG, ADFI and FCR of broiler chickens from d 1 to 14 in response to arginine supplementation, α toxin challenge and their interaction (*P* > 0.05). There was no significance observed in the body weight of d 14 (*P* > 0.05). At 21 d of age, the α toxin challenge significantly decreased the body weight (*P* = 0.001), while arginine supplementation significantly increased the body weight (*P* < 0.05). The α toxin challenge significantly decreased the ADG and ADFI of broiler chickens from d 15 to 21 (*P* < 0.05), while the addition of arginine significantly increased the ADFI (*P* < 0.05). The FCR from d 15 to 21 was not affected by arginine supplementation, α toxin challenge and their interaction (*P* > 0.05).

**Table 1:** Effects of arginine supplementation on growth performance of broiler chickens.

Items	1-14d				15-21d			
	ADG, g	ADFI, g	FCR	BW, g	ADG, g	ADFI, g	FCR	BW, g
CON	20.58	30.16	1.47	328.04	44.82	65.76	1.47	603.33
ARG	20.56	30.57	1.49	342.29	45.94	69.30	1.51	619.38
ATX	20.39	30.09	1.51	323.28	42.08	61.76	1.44	565.42
ARG+ATX	20.39	29.57	1.45	335.18	42.42	64.06	1.52	587.19
SEM	0.187	0.484	0.021	3.838	0.455	0.794	0.017	5.600
Main effect								
Arg								
–	20.48	30.12	1.49	325.50	43.45	63.89	1.46	584.38
+	20.48	30.07	1.47	338.46	44.18	66.68	1.51	600.98
α toxin								
–	20.57	30.38	1.48	334.62	45.38	67.53	1.49	611.35
+	20.39	29.83	1.48	328.83	42.25	62.99	1.48	577.86
*P* value								
Arg	0.984	0.954	0.709	0.100	0.332	0.034	0.100	0.041
α toxin	0.651	0.599	0.964	0.445	<0.001	0.002	0.764	0.001
Interaction	0.989	0.650	0.373	0.879	0.602	0.640	0.653	0.746

CON, birds received a basal diet. ARG, birds received a basal diet supplemented with 0.3% arginine. ATX, birds received a basal diet and subjected to a C. perfringens challenge. ARG+ATX, birds received a basal diet supplemented with 0.3% arginine and subjected to a C. perfringens challenge. ADG, average daily gain. ADFI, average daily feed intake. FCR, feed conversion ratio. BW, body weight. Eight replicates per group.

### Arginine attenuated α toxin-induced reduction in serum immunoglobulins levels in broiler chickens

As presented in **Table 2**, the α toxin challenge significantly decreased serum IgA and IgG levels (*P* < 0.05), which were increased by arginine supplementation (*P* < 0.05). The serum IgM level was not affected by arginine supplementation, α toxin challenge and their interaction (*P* > 0.05).

**Table 2:** Effects of arginine supplementation on serum immunoglobulin (Ig) levels of broiler chickens.

Items	IgA, μg/mL	IgG, μg/mL	IgM, μg/mL
CON	27.59	40.26	3.12
ARG	39.72	41.45	3.12
ATX	24.77	37.12	3.04
ARG+ATX	32.69	40.48	3.08
SEM	1.617	0.495	0.031
Main effect			
Arg			
–	26.18	38.69	3.08
+	35.88	40.9	3.10
α toxin			
–	33.66	40.85	3.12
+	29.09	39.04	3.06
*P* value			
Arg	<0.001	0.011	0.768
α toxin	0.043	0.021	0.351
Interaction	0.364	0.199	0.691

CON, birds received a basal diet. ARG, birds received a basal diet supplemented with 0.3% arginine. ATX, birds received a basal diet and subjected to a C. perfringens challenge. ARG+ATX, birds received a basal diet supplemented with 0.3% arginine and subjected to a C. perfringens challenge. Eight replicates per group.

### Arginine mitigated α toxin-induced intestinal morphology injury in broiler chickens

**Figure 1:**
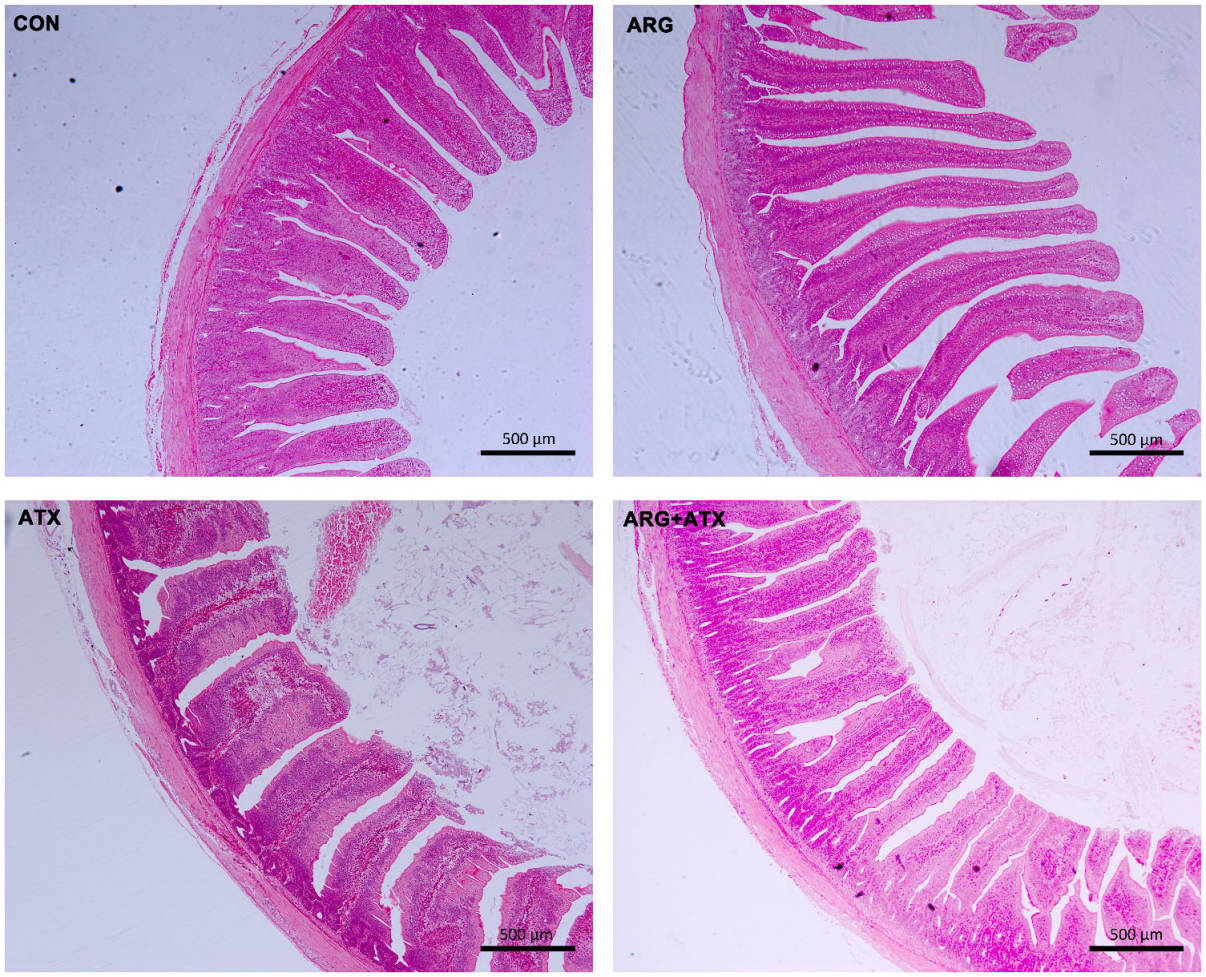
Representative histomorphology pictures of jejunum in broiler chickens. CON, birds received a basal diet. ARG, birds received a basal diet supplemented with 0.3% arginine. ATX, birds received a basal diet and subjected to a *C. perfringens* challenge. ARG+ATX, birds received a basal diet supplemented with 0.3% arginine and subjected to a *C. perfringens* challenge. Original magnification, 50×. Scale bar = 500 μm.

**Figure 1** illustrates that the intestinal structure was nearly normal in the CON and ARG groups, while severely disrupted in the ATX group, as indicated by villi atrophy, villus tip loss, capillary hemorrhages and lymphocytes infiltration. The ARG+ATX group partially alleviated the intestinal morphology damage. **Table 3** demonstrates that the effects of arginine supplementation and α toxin challenge had a significant interaction on the villus height, crypt depth and VCR in the jejunum of broiler chickens (*P* < 0.05). The villus height in the ARG group was the highest among the four groups, with no significant differences observed among the other three groups (*P* > 0.05). Compared with the CON and ARG groups, the ATX group significantly increased the crypt depth (*P* < 0.05). However, compared with the ATX group, the ARG+ATX group significantly reduced the crypt depth (*P* < 0.05). The ARG group had the highest VCR among the four groups, while the ATX group had the lowest. Compared to the ATX group, the ARG+ATX group significantly increased the VCR (*P* < 0.05).

**Table 3:** Effects of arginine supplementation on the jejunal morphology of broiler chickens.

Items	Villus height, μm	Crypt depth, μm	VCR
CON	933.64^b^	113.71^b^	8.45^b^
ARG	1149.76^a^	105.92^b^	10.59^a^
ATX	839.76^b^	152.56^a^	5.98^d^
ARG+ATX	868.02^b^	121.11^b^	7.09^c^
SEM	29.563	4.428	0.377
Main effect			
Arg			
–	894.52	131.64	7.10
+	998.05	113.52	8.71
α toxin			
–	1033.39	110.12	9.62
+	856.24	136.84	6.58
*P* value			
Arg	0.003	0.002	<0.001
α toxin	<0.001	<0.001	<0.001
Interaction	0.019	0.048	0.044

^a-d^Means with no common superscript in the same column indicate significant differences for the interaction effect (P < 0.05).

CON, birds received a basal diet. ARG, birds received a basal diet supplemented with 0.3% arginine. ATX, birds received a basal diet and subjected to a C. perfringens challenge. ARG+ATX, birds received a basal diet supplemented with 0.3% arginine and subjected to a C. perfringens challenge. VCR, villus height-to-crypt depth ratio. Eight replicates per group.

### Arginine alleviated α toxin-induced intestinal inflammatory response and upregulated the mRNA expression of mTOR and SLC38A9 in broiler chickens

As presented in **Table 4**, the mRNA expression levels of *interleukin* (*IL)-1β*, *IL-6*, *IL-8*, and *IL-17* was significantly increased by the α toxin challenge (*P* < 0.05). Arginine supplementation significantly reduced the mRNA expression levels of *IL-1β*, *IL-6*, and *IL-17* (*P* < 0.05). The mRNA expression of *mTOR* and *SLC38A9* was significantly decreased by the α toxin challenge (*P* < 0.05), while arginine supplementation significantly increased *mTOR* mRNA expression (*P* < 0.05).

**Table 4:** Effects of arginine supplementation on the mRNA expression of inflammatory cytokines, mTOR and SLC38A9 of broiler chickens.

Items	*IL-1β*	*IL-6*	*IL-8*	*IL-10*	*IL-17*	*TNF-α*	*mTOR*	*SLC38A9*
CON	1.02	1.06	1.34	1.05	0.99	0.97	1.01	1.01^b^
ARG	0.70	0.69	0.94	1.45	0.88	0.87	1.13	1.42^a^
ATX	1.57	1.91	1.92	0.73	1.57	1.19	0.89	0.81^c^
ARG+ATX	0.90	1.21	1.59	1.09	0.97	0.94	1.00	0.68^c^
SEM	0.089	0.132	0.139	0.122	0.091	0.054	0.030	0.058
Main effect								
Arg								
–	1.31	1.48	1.63	0.89	1.28	1.08	0.96	0.91
+	0.80	0.97	1.27	1.27	0.93	0.91	1.07	1.05
α toxin								
–	0.86	0.88	1.14	1.25	0.94	0.92	1.06	1.21
+	1.26	1.53	1.76	0.91	1.25	1.06	0.94	0.75
*P* value								
Arg	0.008	0.004	0.161	0.121	0.043	0.173	0.037	0.007
α toxin	0.001	0.021	0.023	0.160	0.034	0.101	0.030	<0.001
Interaction	0.177	0.445	0.885	0.926	0.129	0.489	0.884	<0.001

^a-c^Means with no common superscript in the same column indicate significant differences for the interaction effect (P < 0.05).

CON, birds received a basal diet. ARG, birds received a basal diet supplemented with 0.3% arginine. ATX, birds received a basal diet and subjected to a C. perfringens challenge. ARG+ATX, birds received a basal diet supplemented with 0.3% arginine and subjected to a C. perfringens challenge. TNF-α, tumor necrosis factor alpha. mTOR, mechanistic target of rapamycin. SLC38A9, solute carrier family 38 member 9. Eight replicates per group.

### Arginine alleviated the decrease in cell viability and the increase in cytotoxicity induced by α toxin in IEC-6 cells

**Figure 2:**
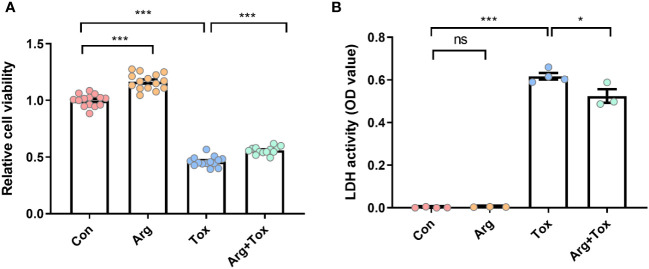
Effects of arginine on cell viability and LDH release in IEC-6 cells cultured with α toxin. **(A)** cell viability in IEC-6 cells. **(B)** LDH activity in the culture supernatant of IEC-6 cells. Con, cells cultured in arginine-free DMEM. Arg, cells cultured with 4 mM arginine. Tox, cells cultured with 50 U/L α toxin. Arg+Tox, cells cultured with 4 mM arginine for 24 h and then cultured with both 50 U/L α toxin and 4 mM arginine for another 4 h. Data are expressed as means ± SEM. Significance was set at *P* < 0.05. *P < 0.05, ***P < 0.001, and ns means no significant.

**Figure 2A** shows that treatment with α toxin challenge alone caused a significant decrease in cell viability compared with the Con group (*P* < 0.001). However, the Arg+Tox group significantly increased the cell viability compared to the Tox group (*P* < 0.001). As shown in **Figure 2B**, compared to the Con group, the Tox group significantly elevated the release of LDH from IEC-6 cells (*P* < 0.001), indicating an increase in cytotoxicity. However, the Arg+Tox group significantly reduced this cytotoxicity (*P* < 0.05).

### Arginine inhibited α toxin-induced cell apoptosis in IEC-6 cells

**Figure 3:**
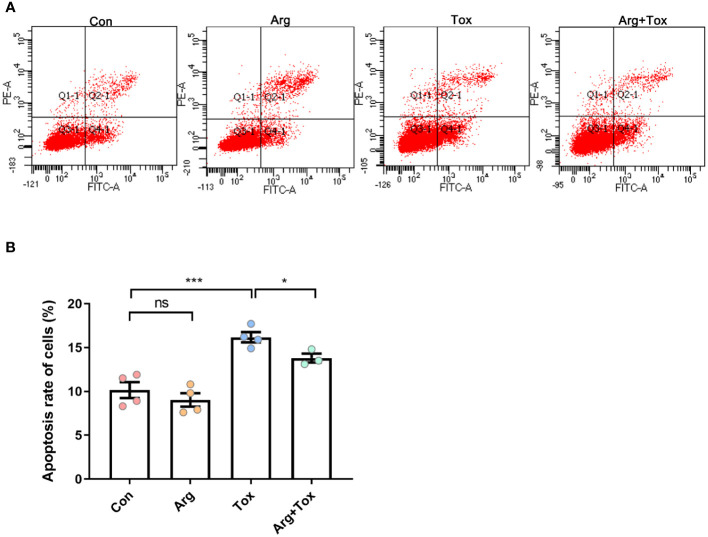
Effects of arginine on apoptosis rate in IEC-6 cells cultured with α toxin determined by flow cytometry. **(A)** flow cytometry following FITC-Annexin V and PI double staining. **(B)** apoptosis rate in IEC-6 cells. Con, cells cultured in arginine-free DMEM. Arg, cells cultured with 4 mM arginine. Tox, cells cultured with 50 U/L α toxin. Arg+Tox, cells cultured with 4 mM arginine for 24 h and then cultured with both 50 U/L α toxin and 4 mM arginine for another 4 h. Data are expressed as means ± SEM. Significance was set at *P* < 0.05. *P < 0.05, ***P < 0.001, and ns means no significant.

As illustrated in **Figure 3**, compared to the Con group, treatment with α toxin alone significantly increased the apoptosis rate of cells (*P* < 0.001). However, the Arg+Tox group led to a reduction in the apoptosis rate of cells compared with the Tox group (*P* < 0.05).

**Figure 4:**
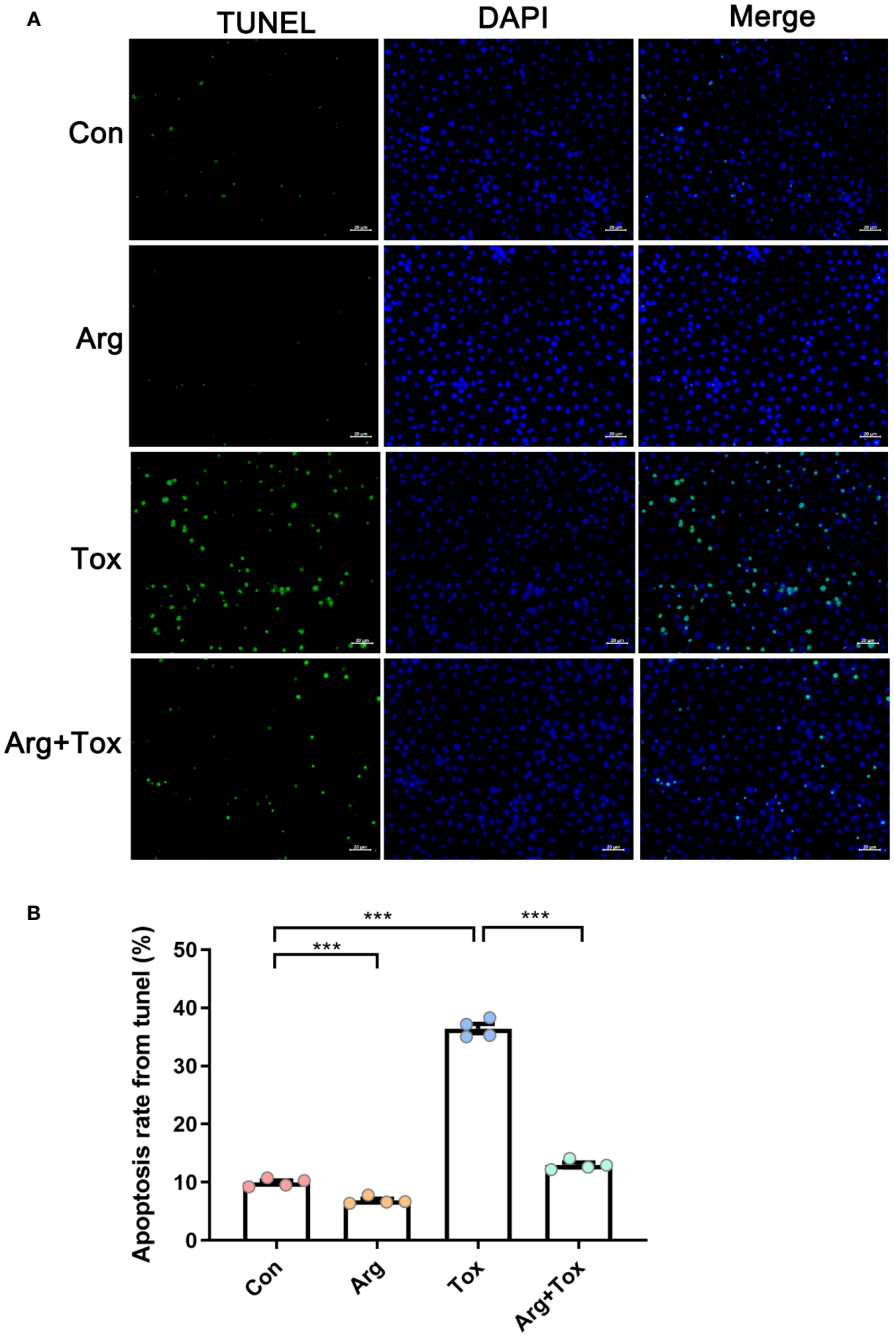
Effects of arginine on apoptosis rate in IEC-6 cells cultured with α toxin determined by TUNEL assay. **(A)** immunofluorescence staining of IEC-6 cells. The scale bar represents 20 μm. **(B)** apoptosis rate in IEC-6 cells. Con, cells cultured in arginine-free DMEM. Arg, cells cultured with 4 mM arginine. Tox, cells cultured with 50 U/L α toxin. Arg+Tox, cells cultured with 4 mM arginine for 24 h and then cultured with both 50 U/L α toxin and 4 mM arginine for another 4 (h) Data are expressed as means ± SEM. Significance was set at *P* < 0.05. ***P < 0.001.

As depicted in **Figure 4**, TUNEL assays indicated that arginine-treated cells without α toxin challenge had lower cell apoptosis rate than the Con group (*P* < 0.001). However, the Tox group significantly increased the cell apoptosis rate compared to the Con group (*P* < 0.001). Pretreatment with arginine in the challenged cells resulted in a reduction of cell apoptosis (*P* < 0.001).

### Arginine suppressed the increased mRNA expression of inflammation and apoptosis-related genes in IEC-6 cells induced by α toxin

**Figure 5:**
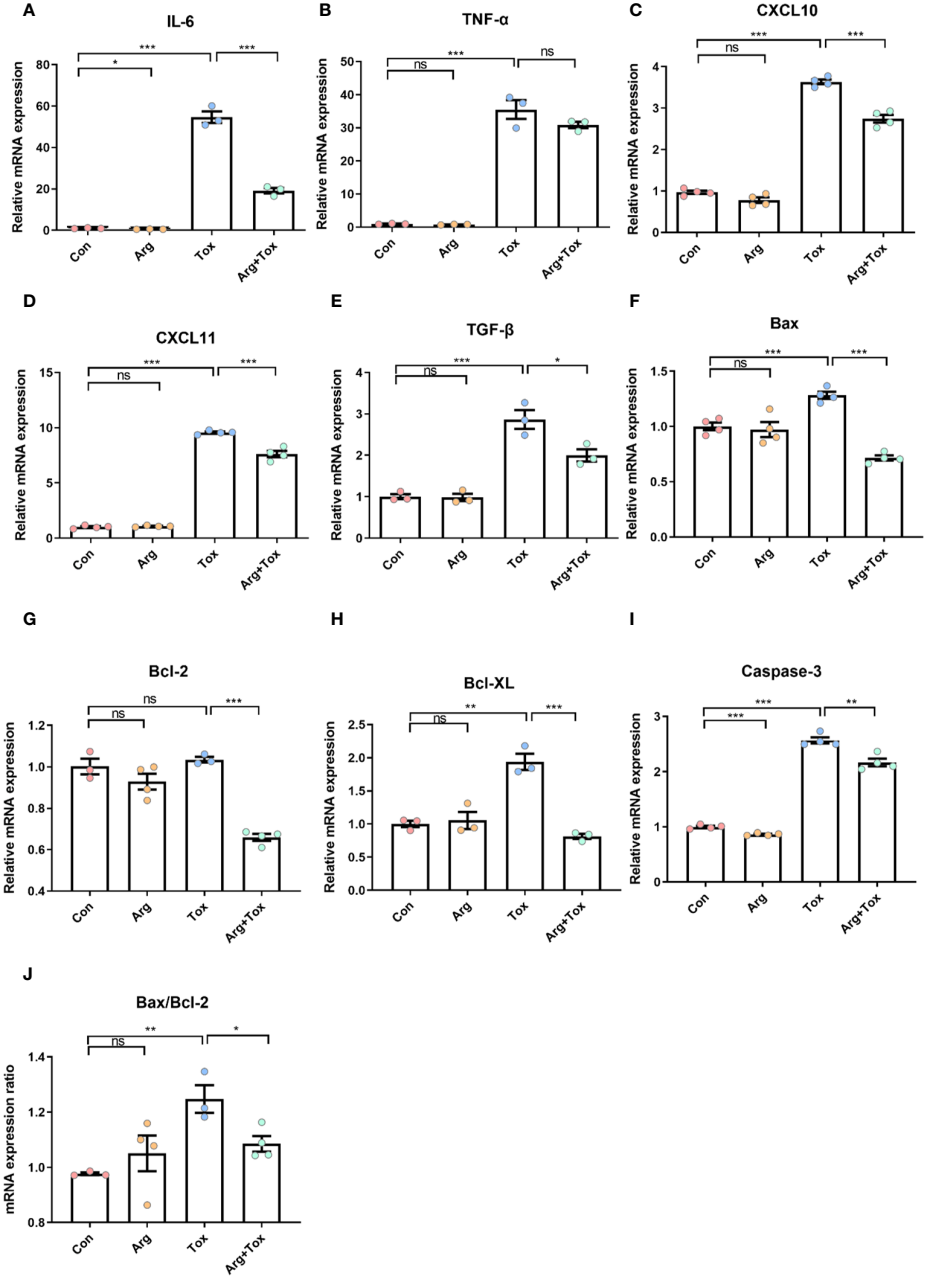
Effects of arginine on the mRNA expression of inflammatory and apoptotic genes in IEC-6 cells cultured with α toxin. **(A–E)** mRNA expression of inflammatory genes *IL-6*, *TNF-α*, *CXCL10*, *CXCL11* and *TGF-β* in IEC-6 cells. **(F–I)** mRNA expression of apoptotic genes *Bax*, *Bcl-2*, *Bcl-XL* and *Caspase-3* in IEC-6 cells. **(J)** Bax/Bcl-2 ratio in IEC-6 cells. Con, cells cultured in arginine-free DMEM. Arg, cells cultured with 4 mM arginine. Tox, cells cultured with 50 U/L α toxin. Arg+Tox, cells cultured with 4 mM arginine for 24 h and then cultured with both 50 U/L α toxin and 4 mM arginine for another 4 h. Data are expressed as means ± SEM. Significance was set at *P* < 0.05. *P < 0.05, **P < 0.01, ***P < 0.001, and ns means no significant.

As presented in **Figures 5A–E**, when compared to the Con and Arg groups, the Tox group significantly increased the mRNA expression levels of pro-inflammatory cytokines *IL-6* and *tumor necrosis factor alpha (TNF-α)*, as well as chemokines *C-X-C motif chemokine ligand 10* (*CXCL10)* and *C-X-C motif chemokine ligand 11* (*CXCL-11)*, along with anti-inflammatory cytokines *transforming growth factor-β* (*TGF-β)* (*P* < 0.001). However, pretreatment with arginine in cells cultured with α toxin significantly reduced the mRNA expression levels of *IL-6*, *CXCL-10*, *CXCL-11* and *TGF-β*, when compared to cells treated with α toxin challenge alone (*P* < 0.05).

**Figures 5F–J** shows that compared to the Con group, the α toxin challenge alone significantly increased the mRNA expression levels of pro-apoptotic factors *B-cell lymphoma-2 associated X protein (Bax)* and *cysteinyl aspartate specific proteinase 3 (Caspase-3)*, as well as anti-apoptotic factors *B-cell lymphoma-extra large (Bcl-XL)* (*P* < 0.01). Pretreatment with arginine prior to α toxin challenge significantly reduced the mRNA expression levels of *Bax*, *B-cell lymphoma-2 (Bcl-2)*, *Bcl-XL* and *Caspase-3* (*P* < 0.01), compared to cells subjected to α toxin challenge alone. In addition, the α toxin challenge significantly elevated the *Bax/Bcl-2* ratio (*P* < 0.01), and these effects were effectively reversed by pretreatment with arginine prior to the toxin challenge (*P* < 0.05).

### Arginine upregulated the mRNA expression of genes in SLC38A9/mTORC1 pathway in α toxin-treated IEC-6 cells

**Figure 6:**
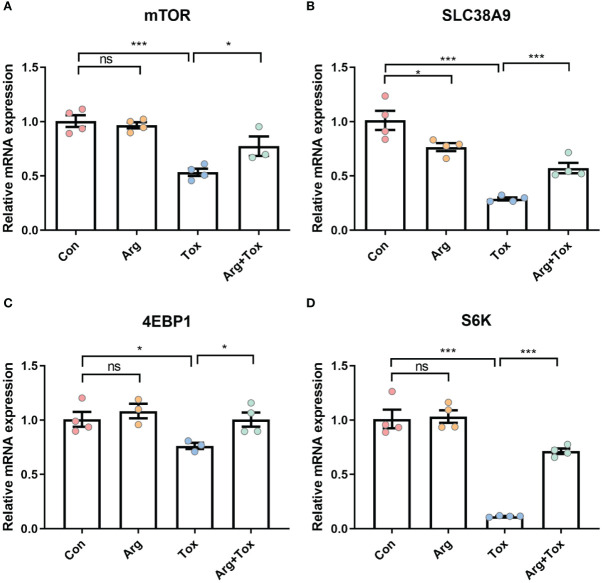
Effects of arginine on the mRNA expression of genes in SLC38A9/mTORC1 pathway in IEC-6 cells cultured with α toxin. **(A–D)** mRNA expression of inflammatory-related genes *mTOR*, *SLC38A9*, *4EBP1* and *S6K* in IEC-6 cells. Con, cells cultured in arginine-free DMEM. Arg, cells cultured with 4 mM arginine. Tox, cells cultured with 50 U/L α toxin. Arg+Tox, cells cultured with 4 mM arginine for 24 h and then cultured with both 50 U/L α toxin and 4 mM arginine for another 4 h. Data are expressed as means ± SEM. Significance was set at *P* < 0.05. *P < 0.05, ***P < 0.001, and ns means no significant.

Compared with cells in the Con group, the mRNA expression levels of *mTOR* and *SLC38A9* were significantly decreased in the Tox group (*P* < 0.001, **Figures 6A, B**). However, these reductions were mitigated in the Arg+ Tox group (*P* < 0.05). Compared with the Con and Arg groups, the cells exposed to the α toxin alone exhibited lower mRNA expression levels of *4EBP1* (*P* < 0.05, **Figures 6C, D**) and *S6K* (*P* < 0.001). However, pretreatment with arginine in the α toxin-treated cells led to an increase in the mRNA expression of these genes (*P* < 0.05).

### Arginine exhibited no protective effects on IEC-6 cells treated with mTOR and SLC38A9 siRNA

**Figure 7:**
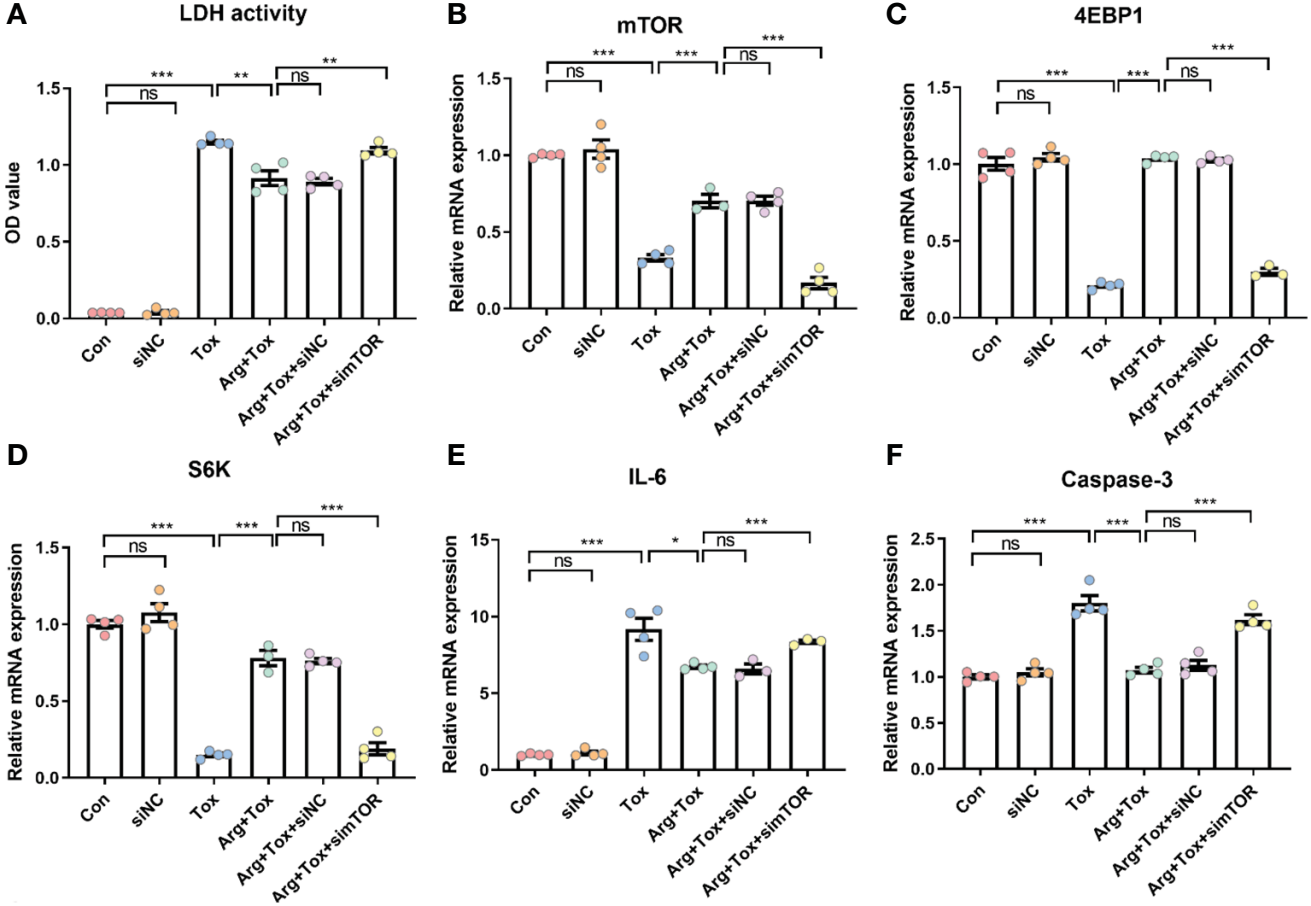
Effects of arginine on IEC-6 cells cultured with α toxin after silencing mTOR. **(A)** LDH activity in the culture supernatant of IEC-6 cells. **(B–D)** mRNA expression of *mTOR*, *4EBP1* and *S6K* in IEC-6 cells. **(E, F)** mRNA expression of inflammatory cytokines *IL-6* and apoptotic genes *Caspase-3* in IEC-6 cells. Con, cells cultured in arginine-free DMEM. siNC, cells cultured with NC siRNA. Tox, cells cultured with 50 U/L α toxin. Arg+Tox, cells cultured with 4 mM arginine for 24 h and then cultured with both 50 U/L α toxin and 4 mM arginine for another 4 h. Arg+Tox+siNC, cells transfected with NC siRNA and then pretreated with arginine for 24 h prior to incubation with both arginine and α toxin for another 4 h. Arg+Tox+simTOR, cells transfected with mTOR siRNA and then pretreated with arginine for 24 h prior to incubation with both arginine and α toxin for another 4 h. Data are expressed as means ± SEM. Significance was set at *P* < 0.05. *P < 0.05, **P < 0.01, ***P < 0.001, and ns means no significant.

Arginine pretreatment markedly decreased the α toxin-induced cytotoxicity, while mTOR silencing significantly attenuated the effect of arginine (*P* < 0.01, **Figures 7A, B**). Moreover, arginine-induced upregulation of *4EBP1* and *S6K* mRNA expression downstream of mTORC1 was inhibited by mTOR silencing (*P* < 0.001, **Figures 7C, D**). The decline in mRNA expression of pro-inflammatory (*IL-6*) and pro-apoptotic (*Caspase-3*) genes due to arginine treatment was abrogated by mTOR silencing (*P* < 0.001, **Figures 7E, F**).

**Figure 8:**
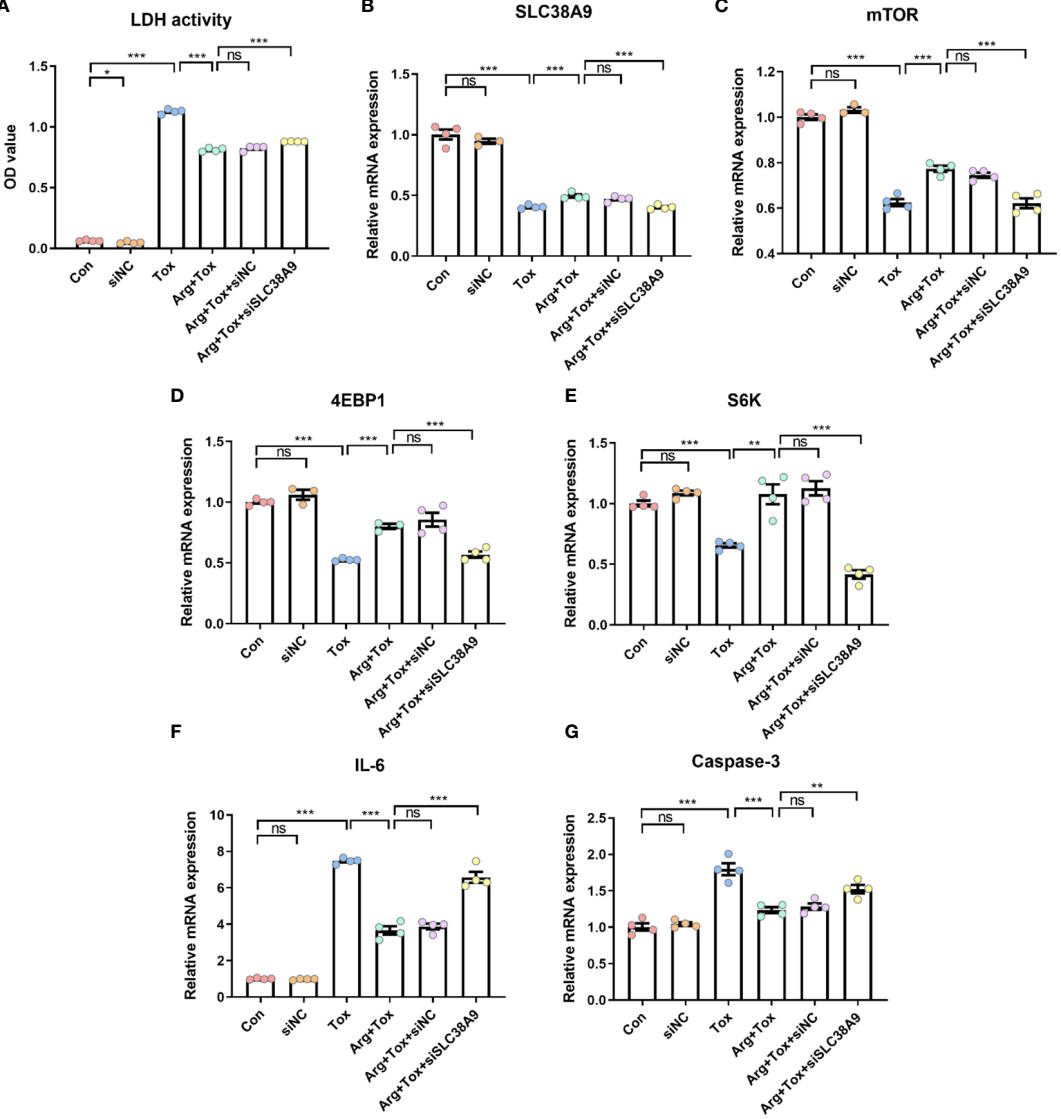
Effects of arginine on IEC-6 cells cultured with α toxin after silencing SLC38A9. **(A)** LDH activity in the culture supernatant of IEC-6 cells. **(B–E)** mRNA expression of *SLC38A9*, *mTOR*, *4EBP1* and *S6K* in IEC-6 cells. **(F, G)** mRNA expression of inflammatory cytokines *IL-6* and apoptotic genes *Caspase-3* in IEC-6 cells. Con, cells cultured in arginine-free DMEM. siNC, cells cultured with NC siRNA. Tox, cells cultured with 50 U/L α toxin. Arg+Tox, cells cultured with 4 mM arginine for 24 h and then cultured with both 50 U/L α toxin and 4 mM arginine for another 4 h. Arg+Tox+siNC, cells transfected with NC siRNA and then pretreated with arginine for 24 h prior to incubation with both arginine and α toxin for another 4 h. Arg+Tox+siSLC38A9, cells transfected with SLC38A9 siRNA and then pretreated with arginine for 24 h prior to incubation with both arginine and α toxin for another 4 h. Data are expressed as means ± SEM. Significance was set at *P* < 0.05. *P < 0.05, **P < 0.01, ***P < 0.001, and ns means no significant.

SLC38A9 silencing significantly increased the cytotoxicity of cells cultured with arginine (*P* < 0.001, **Figures 8A, B**). Additionally, SLC38A9 silencing abolished the increased *mTOR* mRNA expression caused by arginine treatment in IEC-6 cells exposed to α toxin (*P* < 0.001, **Figure 8C**). The increase in mRNA expression of *4EBP1* and *S6K* mRNA due to arginine treatment was abrogated by SLC38A9 silencing (*P* < 0.001, **Figures 8D, E**). Knockdown of SLC38A9 also counteracted the decline in mRNA expression of pro-inflammatory (*IL-6*) and pro-apoptotic (*Caspase-3*) genes caused by arginine (*P* < 0.001, **Figures 8F, G**).

## Discussion

Immune stress often results in growth performance impairment and structural gut damage in broiler chickens. In this study, arginine supplementation alleviated the decreased ADFI and final body weight induced by the α toxin challenge, highlighting the importance of exogenous arginine supply during immune stress. Similarly, Liu et al. (27) demonstrated that arginine supplementation decreased body weight loss in pigs challenged with LPS. Arginine may regulate metabolism to promote protein synthesis and reduce protein catabolism during stress and infections (28) by stimulating the secretion of growth hormone, insulin and glucagon (29). Therefore, arginine supplementation alleviated the growth suppression induced by the α toxin challenge, which may be partially due to its influence on protein metabolism. Serum antibody levels are valuable indicators of humoral immunity, with IgA, IgM, and IgG being the main antibodies that selectively bind to antigens (30). In this study, the α toxin challenge lowered the serum IgA and IgG levels of broiler chickens, while arginine supplementation increased these antibody levels. This suggested that arginine pretreatment can enhance Ig levels to counteract the adverse effects of the α toxin challenge in broilers. Similarly, Chen et al. (31) reported that dietary supplementation with 0.3% arginine increased serum IgA and IgG levels in geese.

The morphology of the small intestine, including villus height, crypt depth and VCR, can serve as an indicator of intestinal health and integrity. In this study, arginine supplementation alleviated the increased jejunal crypt depth and decreased VCR of broiler chickens induced by the α toxin, demonstrating its protective effect on intestinal mucosa. Our previous studies also reported that arginine addition reduced the intestinal morphological injury caused by *Salmonella enterica serovar Typhimurium* challenge in broiler chickens (32). In addition, arginine supplementation was found to enhance the jejunal morphology of low-birth-weight piglets (33). Therefore, arginine supplementation alleviated the growth suppression induced by the α toxin challenge partially by alleviating the intestinal mucosa injury. Based on our *in vitro* findings, arginine pretreatment mitigated the increased LDH production and decreased viability of IEC-6 cells induced by α toxin, suggesting that arginine pretreatment could lessen the detrimental effects of α toxin challenge on cells. Furthermore, both flow cytometry and TUNEL staining assays in this study consistently demonstrated that arginine pretreatment effectively prevented the enhanced cell apoptosis generated by the α toxin challenge. The reduction in cytotoxicity and apoptosis in the intestinal epithelial cells may be the possible mechanism by which arginine supplementation mitigated the damage to intestinal morphology induced by the α toxin challenge *in vivo*.

Cytokines and chemokines have been identified as important regulators of inflammation and infection (34). Intestinal epithelial cells produce various proinflammatory cytokines, such as IL-1β, IL-6, TNF-α and IL-17, which play a critical role in intestinal innate immune defense (35). IL-17 induces inflammation by enhancing the synthesis of essential pro-inflammatory cytokines, primarily IL-1β, IL-6 and TNF-α (36). CXCL10 and CXCL11 are proinflammatory chemokines that recruit immune cells, including monocytes and T cells, to the sites of infection or injury (37). The current study found that arginine supplementation significantly reduced the elevation of mRNA expression levels of *IL-1β*, *IL-6* and *IL-17* induced by α challenge in the jejunum of broiler chickens, as well as the mRNA expression of *IL-6*, *CXCL10* and *CXCL11* in the IEC-6 cells. This result suggested that excessive activation of the inflammatory response was inhibited by arginine. The balance between pro- and anti-inflammatory cytokines is crucial for animal health. In the present study, the administration of arginine to IEC-6 cells resulted in a suppression of the elevated transcription of *TGF-β*, an anti-inflammatory cytokine, because of the α toxin challenge. Consistent with our results, Tan et al. (38) observed that the dietary arginine addition could mitigate the upregulation of mRNA expression of pro- and anti-inflammatory cytokines in broiler chickens challenged with lipopolysaccharide. Our previous *in vivo* and *in vitro* studies indicated that arginine can reduce the increased mRNA expression of pro- and anti-inflammatory cytokines in chickens induced by *C. perfringens* infection (14).

Apoptosis is an essential mechanism employed by the immune system to fight infections and eliminate cells with irreversible DNA damage (39). The Bcl-2 family proteins regulate programmed cell death triggered by mitochondrial dysfunction. Some family members, like Bcl-2 and Bcl-XL, inhibit apoptosis, whereas others, like Bax, promote cell death (40). The overexpression of Bax promotes the release of cytochrome C, which subsequently activates the downstream Caspase-3 protease, mediating cell death (41, 42). Bcl-2 and Bcl-XL can heterodimerize with Bax and neutralize the effects of the latter (43). Our results showed that arginine treatment alleviated the elevation of *Bax* and *Caspase-3* mRNA expression caused by α toxin challenge in IEC-6 cells, which indicated that arginine reversed α toxin-induced cell apoptosis. These findings are in line with recent studies demonstrating that arginine effectively suppresses doxorubicin-induced vascular dysfunction by attenuating apoptosis (44). In addition, Ma et al. (45) reported that arginine pretreatment enhanced alveolar integrity and function by reducing LPS-induced apoptosis of alveolar cells, expression of inflammatory cytokines and chemokines, and accumulation of neutrophils and macrophages in the lung tissues of mice.

Arginine promotes diverse physiological effects, including immune cell activation in animals, mostly by activating mTORC1 (18–20). This multi-protein complex consists of mTOR, the regulatory associated protein of mTOR (Raptor), and mammalian LST8 homolog (mLst8) as core components (19). mTORC1 can regulate its downstream targets, 4EBP1 and S6K to control several physiological processes (46). The current results showed that arginine alleviated α toxin-induced decreased mRNA expression of *mTORC1*, *4EBP1* and *S6K* in IEC-6 cells. This finding is consistent with previous studies demonstrating that arginine activated mTOR and its downstream targets 4EBP1 and S6K, which play crucial roles in processes such as protein synthesis (47) and intestinal inflammation (33). Notably, SLC38A9 has been identified as a bona fide arginine sensor for mTORC1 (23). The activation of mTORC1 by arginine requires SLC38A9, which functions to transport and interact with arginine effectively (22, 48). Thus, our results indicated that arginine alleviated the decreased mRNA expression of *SLC38A9* induced by α toxin in IEC-6 cells, indicating the interconnected relationship between arginine, SLC38A9 and mTORC1 signaling pathways within the cellular context.

To further identify the role of SLC38A9 and mTORC1 in the protective effects of arginine supplementation on cells against α toxin challenge, we employed siRNA to knock down SLC38A9 and mTORC1. Our experimental results revealed that the positive effects of arginine on cytotoxicity, inflammation, and apoptosis induced by α toxin challenge were abolished due to siRNA-mediated knockdown of mTOR in IEC-6 cells. Similarly, the protective effects of arginine against α toxin-induced cytotoxicity, inflammation, and apoptosis were abolished by the knockdown of SLC38A9 in IEC-6 cells. In addition, SLC38A9 silencing weakened the increased *mTOR* mRNA expression caused by arginine treatment in α toxin-treated IEC-6 cells. These results indicated that SLC38A9/mTORC1 pathway played an important role in mediating the cytoprotective effects of arginine against α toxin-induced damage in IEC-6 cells.

In conclusion, our study demonstrated that the immunomodulatory potential of arginine administration in mitigating α toxin-induced challenges both *in vivo* and *in vitro*. Arginine administration effectively attenuated α toxin-induced intestinal injury, which is closely associated with the activation of the SLC38A9/mTORC1 pathway.

## Data availability statement

The original contributions presented in the study are included in the article/**Supplementary Material**. Further inquiries can be directed to the corresponding author.

## Ethics statement

The animal study was approved by Animal Care and Use Committee of Qingdao Agricultural University (No. DKY20220905). The study was conducted in accordance with the local legislation and institutional requirements.

## Author contributions

XW: Methodology, Data curation, Writing – original draft, Investigation. TZ: Methodology, Data curation, Writing – original draft, Investigation. WL: Supervision, Project administration, Writing – review & editing. HW: Resources, Methodology, Writing – review & editing. LY: Resources, Investigation, Writing – review & editing. XZ: Software, Methodology, Writing – review & editing. LZ: Software, Methodology, Writing – review & editing. NW: Software, Methodology, Writing – review & editing. BZ: Supervision, Project administration, Investigation, Funding acquisition, Writing – review & editing.

## Glossary

**Table:**

mTORC1	mechanistic target of rapamycin complex 1
SLC38A9	solute carrier family 38 member 9
4EBP1	eukaryotic translation initiation factor 4E (eIF4E)-binding protein 1
S6K	ribosomal protein S6 kinase
GAPDH	glyceraldehyde-3-phosphate dehydrogenase
ADG	average daily gain
ADFI	average daily feed intake
FCR	feed conversion ratio
Ig	immunoglobulins
VCR	villus height-to-crypt depth ratio
DMEM	Dulbecco’s Modified Eagle Medium
siRNA	small interfering RNA
CCK-8	Cell Counting Kit-8
LDH	lactic dehydrogenase
PI	propidium iodide
SEM	standard error of the mean
TNF-α	tumor necrosis factor alpha
CXCL10	C-X-C motif chemokine ligand 10
CXCL11	C-X-C motif chemokine ligand 11
TGF-β	transforming growth factor-β
Bax	B-cell lymphoma-2 associated X protein
Bcl-2	B-cell lymphoma-2
Bcl-XL	B-cell lymphoma-extra large
Caspase-3	cysteinyl aspartate specific proteinase 3
