# Supplementary material for: Arginine alleviates Clostridium perfringens α toxin-induced intestinal injury in vivo and in vitro via the SLC38A9/mTORC1 pathway
Source: Front Immunol. 2024 Apr 4;15:1357072. doi: 10.3389/fimmu.2024.1357072 (PMC11024335; doi:10.3389/fimmu.2024.1357072)
Supplement: Supplementary file 1 [file Table_1.docx]

Supplementary Material

**Table S1** Composition and nutrient levels of basal diet (air-dry basis)

| Ingredients (%, unless otherwise indicated) | Basal diet | Arginine diet |
| --- | --- | --- |
| Corn | 62.69 | 62.69 |
| Soybean meal, 46% CP | 26.00 | 26.00 |
| Corn gluten meal | 4.50 | 4.50 |
| Soybean oil | 1.80 | 1.80 |
| Limestone | 1.25 | 1.25 |
| Calcium hydrophosphate | 2.10 | 2.10 |
| L-Lys-HCL | 0.23 | 0.23 |
| D,L-Met, 98% | 0.16 | 0.16 |
| Choline chloride, 70% | 0.10 | 0.10 |
| NaCl | 0.30 | 0.30 |
| Zeolite powder | 0.00 | 0.31 |
| L-Ala | 0.61 | 0.00 |
| L-Arg | 0.00 | 0.30 |
| Mineral premix*^a^* | 0.15 | 0.15 |
| Vitamin premix*^b^* | 0.10 | 0.10 |
| Ethoxyquin, 60% | 0.01 | 0.01 |
| Total | 100.00 | 100.00 |
| Nutrition level*^c^* |  |  |
| ME, MJ/kg | 12.59 | 12.58 |
| CP | 20.73 | 20.73 |
| Ca | 1.02 | 1.02 |
| Non-phytate phosphorus | 0.45 | 0.45 |
| Lys | 1.16 | 1.16 |
| Met | 0.51 | 0.51 |
| Arg | 1.21 | 1.51 |

*^a^*Provided per kilogram of complete diet: Mn, 100 mg; Fe, 80 mg; Zn, 75 mg; Cu, 8 mg; I, 0.4 mg; and Se, 0.3 mg

*^b^*Provided per kilogram of complete diet: vitamin A, 8,000 IU; vitamin D_3_, 2500 IU; vitamin E, 20 IU; vitamin K_3_, 2 mg; vitamin B_1_, 2 mg; vitamin B_2_, 6 mg; vitamin B_6_, 4.5mg; pantothenic acid, 12 mg; vitamin B_12_, 0.02 mg; niacin, 50 mg; folic acid, 1 mg and biotin, 0.15 mg

*^c^*Calculated value.

**Table S2** Sequences of primers used for quantitative real-time PCR in broiler chickens

| Gene name*^a^* | Accession number | Primer sequence*^b^* (5’ to 3’) | Product size (bp) | Efficiency (%) |
| --- | --- | --- | --- | --- |
| *IL-1β* | XM_015297469.1 | F: ACTGGGCATCAAGGGCTA | 131 | 104.4 |
|  |  | R: GGTAGAAGATGAAGCGGGTC |  |  |
| *IL-6* | XM_015281283.1 | F: CGCCCAGAAATCCCTCCTC | 152 | 102.0 |
|  |  | R: AGGCACTGAAACTCCTGGTC |  |  |
| *IL-8* | XM_015301388.1 | F: ATGAACGGCAAGCTTGGAGCTG | 233 | 120.0 |
|  |  | R: TCCAAGCACACCTCTCTTCCATCC |  |  |
| *IL-10* | NM_001004414.2 | F: CGCTGTCACCGCTTCTTCA | 88 | 98.4 |
|  |  | R: TCCCGTTCTCATCCATCTTCTC |  |  |
| *IL-17* | NM_204460.1 | F: CTCCGATCCCTTATTCTCCTC | 292 | 97.0 |
|  |  | R: AAGCGGTTGTGGTCCTCAT |  |  |
| *TNF-α* | XM_046927265.1 | F: GAGCGTTGACTTGGCTGTC | 64 | 95.8 |
|  |  | R: AAGCAACAACCAGCTATGCAC |  |  |
| *mTOR* | XM_417614.8 | F: GGAGCAGCAAGAAGAGTC | 92 | 93.7 |
|  |  | R: AAGGATGGTGAGGAGGAAT |  |  |
| *SLC38A9* | XM_046905524.1 | F: GTATCCGTCGCTCATCTAC | 106 | 103.8 |
|  |  | R: CAGATTAGCCAGTCCAAGG |  |  |
| *GAPDH* | NM_204305.1 | F: TGCTGCCCAGAACATCATCC | 142 | 98.2 |
|  |  | R: ACGGCAGGTCAGGTCAACAA |  |  |

*^a^**IL*, interleukin. *TNF-α*, tumor necrosis factor alpha. *mTOR*, mechanistic target of rapamycin. *SLC38A9*, solute carrier family 38 member 9. *GAPDH*, glyceraldehyde-3-phosphate dehydrogenase.

*^b^*F, forward; R, reverse.

**Table S3** Sequences of primers used for quantitative real-time PCR in IEC-6 cells

| Gene name*^a^* | Accession number | Primer sequence*^b^* (5’ to 3’) | Product size (bp) | Efficiency (%) |
| --- | --- | --- | --- | --- |
| *IL-6* | NM_012589.2 | F: TCACAAGTCGGAGGCTTA | 101 | 101.2 |
|  |  | R: GCATCATCGCTGTTCATAC |  |  |
| *TNF-α* | NM_012675.3 | F: TGGAACTGGCAGAGGAG | 92 | 93.6 |
|  |  | R: GAGCAGGAATGAGAAGAGG |  |  |
| *CXCL10* | NM_139089.2 | F: GCTGCTGAGTCTGAGTG | 146 | 98.4 |
|  |  | R: CAACATGCGGACAGGATA |  |  |
| *CXCL11* | NM_182952.2 | F: TTCCAGGCTTCGTTATGTT | 85 | 101.0 |
|  |  | R: TCCTTGATTGCTGCCATT |  |  |
| *TGF-β* | NM_021578.2 | F: ATTCCTGGCGTTACCTTG | 116 | 92.9 |
|  |  | R: CTGTATTCCGTCTCCTTGG |  |  |
| *Bax* | NM_017059.2 | F: AGAGGATGATTGCTGATGTG | 90 | 100.4 |
|  |  | R: AGTTGAAGTTGCCGTCTG |  |  |
| *Bcl-2* | NM_016993.2 | F: GGATACTGGAGATGAAGACT | 70 | 103.9 |
|  |  | R: AGGCTGGAAGGAGAAGAT |  |  |
| *Bcl-XL* | XM_039104291.1 | F: TGACCACCTAGAGCCTTG | 149 | 101.5 |
|  |  | R: GAACTACACCAGCCACAG |  |  |
| *Caspase-3* | XM_039094205.1 | F: TTGAGACAGACAGTGGAAC | 83 | 102.7 |
|  |  | R: GGTAGAGTAAGCATACAGGAA |  |  |
| *mTOR* | NM_019906.2 | F: GAAGAAGGTCACTGAGGATT | 84 | 100.6 |
|  |  | R: AAGGAGATAGAACGGAAGAAG |  |  |
| *SLC38A9* | XM_006231939.4 | F: TTACATCGGACACAACTACC | 83 | 102.1 |
|  |  | R: AAGGAGACTTGACCATTGC |  |  |
| *4EBP1* | NM_053857.2 | F: CTGATGGAGTGTCGGAAC | 93 | 98.8 |
|  |  | R: AGGCTCATCGCTGGTAG |  |  |
| *S6K* | XM_039110841.1 | F: GTACACCATCAGCAAGACT | 123 | 100.4 |
|  |  | R: TCGCATATTCGTAGGCATT |  |  |
| *GAPDH* | NM_017008.4 | F: CCATTCTTCCACCTTTGATGCT | 98 | 99.7 |
|  |  | R: TGTTGCTGTAGCCATATTCATTGT |  |  |

*^a^IL*, interleukin. *TNF-α*, tumor necrosis factor alpha. *CXCL10*, C-X-C motif chemokine ligand 10. *CXCL11*, C-X-C motif chemokine ligand 11. *TGF-β*, transforming growth factor-β. *Bax*, B-cell lymphoma-2 associated X protein. *Bcl-2*, B-cell lymphoma-2. *Bcl-XL*, B-cell lymphoma-extra large. *Caspase-3*, cysteinyl aspartate specific proteinase 3. *mTOR*, mechanistic target of rapamycin. *SLC38A9*, solute carrier family 38 member 9. *4EBP1*, eukaryotic translation initiation factor 4E (eIF4E)-binding protein 1. *S6K*, ribosomal protein S6 kinase. *GAPDH*, glyceraldehyde-3-phosphate dehydrogenase.

*^b^*F, forward; R, reverse.
